# Unravelling the Efficient Photocatalytic Activity of Boron-induced Ti^3+^ Species in the Surface Layer of TiO_2_

**DOI:** 10.1038/srep34765

**Published:** 2016-10-06

**Authors:** Ningdong Feng, Fen Liu, Min Huang, Anmin Zheng, Qiang Wang, Tiehong Chen, Gengyu Cao, Jun Xu, Jie Fan, Feng Deng

**Affiliations:** 1 State Key Laboratory of Magnetic Resonance and Atomic and Molecular Physics, National Center for Magnetic Resonance in Wuhan, Wuhan Institute of Physics and Mathematics, Chinese Academy of Sciences, Wuhan 430071, China; 2 Key Laboratory of Functional Polymer Materials of MOE, Department of Materials Chemistry, Nankai University, Tianjin 300071, P. R. China; 3 Key Laboratory of Applied Chemistry of Zhejiang Province and Department of Chemistry, Zhejiang University, Hangzhou 310027, China

## Abstract

Ti^3+^ species are highly unstable in air owing to their facile oxidation into Ti^4+^ species, and thus they cannot concentrate in the surface layer of TiO_2_ but are mainly present in its bulk. We report generation of abundant and stable Ti^3+^ species in the surface layer of TiO_2_ by boron doping for efficient utilization of solar irradiation. The resultant photocatalysts (denoted as B-TiO_2−x_) exhibit extremely high and stable solar-driven photocatalytic activity toward hydrogen production. The origin of the solar-light activity enhancement in the B-TiO_2−x_ photocatalysts has been thoroughly investigated by various experimental techniques and density functional theory (DFT) calculations. The unique structure invoked by presence of sufficient interstitial boron atoms can lead to substantial variations in density of states of B-TiO_2−x_, which not only significantly narrow the band gap of TiO_2_ to improve its visible-light absorption, but also promote the photogenerated electron mobility to enhance its solar-light photocatalytic activity.

Driven by the decrease of fossil fuel resources and the environmental concerns, the search for new clean and renewable energy technologies is urgent on the research agendas of many research and development communities. In particular, utilization of solar energy for hydrogen production and water/air decontamination has attracted extensive research interest. Since the pioneered discovery of photocatalytic water-splitting on titanium dioxide (TiO_2_)^1^, this semiconductor photocatalysts has been extensively studied and recognized as one of the most promising photocatalysts. However, the wide band gap of pure TiO_2_ (ca. 3.2 eV) renders it only active in the ultraviolet irradiation range. In order to significantly enhance its solar-driven photocatalytic efficiency, band gap engineering is highly desirable not only to improve the solar light harvesting of TiO_2_, but also to suppress the rapid combination of photogenerated electrons and holes. A widely used strategy is to dope TiO_2_ with metal^2^,^3^,^4^ or nonmetal^5^,^6^,^7^,^8^,^9^ elements, which can introduce impurity states into the band gap of TiO_2_ to enhance its visible-light adsorption. However, such an approach possesses significant limitations, i.e., the doped TiO_2_ has low thermal stability and the recombination centers of photoinduced carriers are inevitably increased^4^,^5^,^10^. Recently, it was reported that hydrogenated TiO_2_ nanocrystals with disorder in their surface layers could enhance substantially both solar absorption and solar-driven photocatalytic activities^11^.

Self-doped TiO_2_ (denoted as TiO_2−x_), prepared by incorporating Ti^3+^ into TiO_2_ itself, has emerged as an effective approach to improve its photocatalytic activity in water splitting and water/air decontamination under solar-light irradiation^12^,^13^,^14^,^15^,^16^,^17^,^18^,^19^, where the Ti^3+^ site is always considered as an active site. However, it was found that the low concentration of Ti^3+^ species formed in the TiO_2−x_ would produce localized oxygen vacancy states with energies being 0.75 to 1.18 eV below the conduction band minimum of TiO_2_, which tends to deteriorate the electron mobility in the bulk region and eventually reduce the photocatalytic activity^5^,^20^. According to theoretical calculations, a continuous electronic state just below the conduction band minimum of TiO_2_, induced by the sufficiently high Ti^3+^ doping, may leads to a high electron mobility and eventually a high photocatalytic activity of TiO_2−x_^21^. Thus, the sufficiently high Ti^3+^ in TiO_2−x_ should be essential to enhance its photocatalytic activity under solar-light irradiation. Up to now, many strategies, such as heating under vacuum or in reducing gas (i.e. H_2_), combustion, laser irradiation, and high-energy particle (such as electrons or Ar^+^ ions) bombardment^13^,^22^, have been applied to synthesize TiO_2−x_. These methods, started from pure TiO_2_, possess more or less limitations such as multiple steps, harsh synthesis conditions and expensive facilities. Most importantly, the prepared Ti^3+^ species are highly unstable in air owing to the facile oxidation of Ti^3+^ to Ti^4+ ^23, and thus they cannot concentrate in the surface layer but are mainly present in the bulk of TiO_2−x_. Therefore, it is a big challenge to produce stable and sufficiently abundant Ti^3+^ species, especially the surface-layer Ti^3+^, for the application of TiO_2_ with a simple and inexpensive approach.

Herein, we report a novel and simple approach to generate stable and sufficiently abundant Ti^3+^ in TiO_2_ by boron doping via a sol-gel synthesis method. The resultant photocatalyst (denoted as B-TiO_2−x_) exhibits efficient visible and infrared optical absorption and extremely high and stable photocatalytic activity for hydrogen production. To gain insight into the active center structures and plausible mechanisms associated with the photocatalysts, the results from density functional theory (DFT) calculations were correlated to those obtained from experimental studies, such as UV–vis absorption spectroscopy, X-ray photoelectron spectroscopy (XPS), electron spin resonance (ESR) and nuclear magnetic resonance (NMR) spectroscopy etc. In particular, XPS and ESR were employed to detect the variation of the chemical state of B and Ti, while ^11^B solid-state magic-angle-spinning (MAS) NMR was used to identify the local structures of the contingent dopants. As a result, the exceptionally high photocatalytic activity observed for the B-TiO_2−x_ is ascribed to the synergistic effect of the interstitial B and Ti^3+^ species.

## Results and Discussion

### Solar absorption and photocatalytic activity

**Figure 1:**
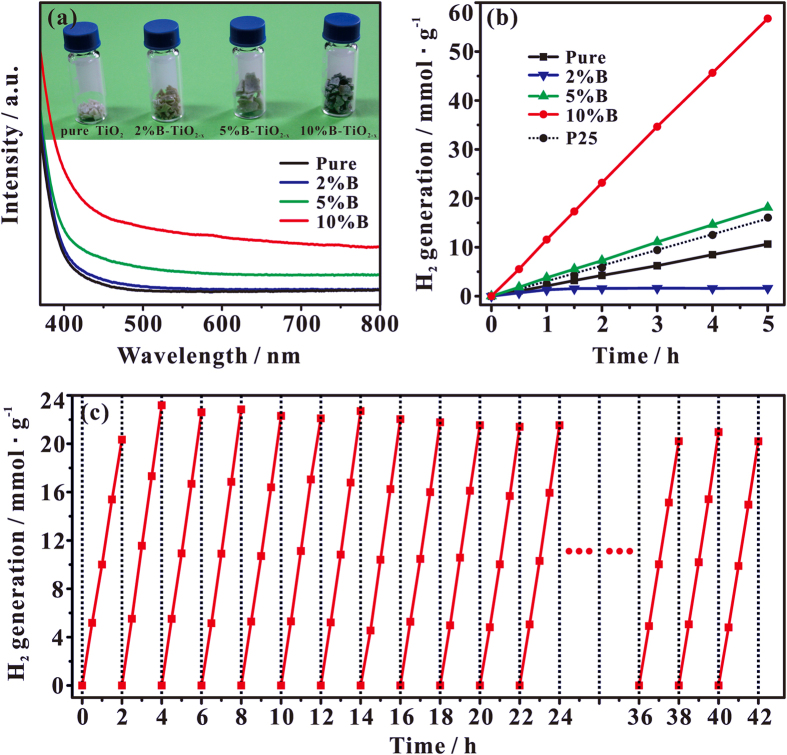
(**a**) UV–Vis absorption spectra of pure TiO_2_ and B-TiO_2−x_ samples with various B-doping content. Inset: a photo comparing pure TiO_2_ and various B-TiO_2−x_ samples. (**b**) The photocatalytic activity of B-TiO_2−x_ and P25 in water splitting hydrogen production under simulated solar-light irradiation. (**c**) Cycling measurements of hydrogen gas generation on 10% B-TiO_2−x_, which were conducted within consecutive 42 hours of overall solar irradiation time.

The optical response of as-prepared pure TiO_2_ and B-TiO_2−x_ with different B doping was evaluated by the diffusive reflectance spectroscopy, as shown in Fig. 1a. The band gap of the B-TiO_2−x_ samples can be derived from the plot of transformed Kubelka-Monk function against photo energy (Figure S1 in Supplementary Information, SI). It can be found that the band gap of pure TiO_2_ is deduced approximately at 3.25 eV, and the band gap of TiO_2_ narrows gradually from 3.20 to 3.0 eV with the amount of doped B increasing from 2 to 10% (Figure S1 in SI). More interestingly, for the B-TiO_2−x_ samples, the intensity of visible-light absorption between 400 and 780 nm (the photo energy between 3.1 and 1.59 eV) increases gradually with the increase of the amount of doped B (Fig. 1a). Especially, for the 10% B-TiO_2−x_ samples, considerable visible-light absorption is still observable at ca. 1350 nm (corresponding to an optical energy at 0.92 eV) in the UV-Vis spectrum (Figure S2 in SI). All these experimental results suggest that rich mid-gap states should exist in the band gap of B-TiO_2−x_ samples, and the B doping is responsible for the high visible-light absorption of B-TiO_2−x_ photocatalysts.

The B-TiO_2−x_ photocatalysts exhibit extremely high solar-driven photocatalytic activity toward H_2_ production from water in the presence of methanol solution (5.0 volume %). Figure 1b shows the time course of H_2_ evolution for the photocatalysts with different B doping. The photocatalytic activity generally increases with increasing the content of dopant. Particularly, when the amount of dopant B is about 10%, the B-TiO_2−x_ catalyst shows the best photocatalytic activity (also see Figure S3 in SI). We found that 1 hour of solar irradiation generates 0.059 mmol of H_2_ using 0.005 g of the 10% B-TiO_2−x_ sample and 0.6% wt. Pt as the co-catalyst (11.8 mmol/hour/g of photocatalyst). The photocatalytic activity of 10% B-TiO_2−x_ is ca. 3.5 times as much as that of a commercial P25. (Fig. 1b).

Besides its excellent activity, as shown in Fig. 1c, the 10% B-TiO_2−x_ photocatalyst also exhibits excellent stability in the photocatalytic production of hydrogen under solar-light irradiation. There is no noticeable decrease in H_2_ production rate for the 10% B-TiO_2−x_ sample in a 21 cycling test within a 42 h photocatalytic reaction period. The energy conversion efficiency, defined as the ratio of the energy of solar-produced H_2_ and the energy of incident sunlight, reaches ca. 21% for the 10% B-TiO_2−x_ sample.

To establish the structure-activity relationship, the detailed structural characteristis of B-TiO_2−x_ photocatalysts was investigated with various analytical techniques, including X-ray photoelectron spectroscopy (XPS), electron spin resonance (ESR), transmission electron microscopy (TEM) and solid-state nuclear magnetic resonance (NMR).

### Chemical states and electronic structure analysis

**Figure 2:**
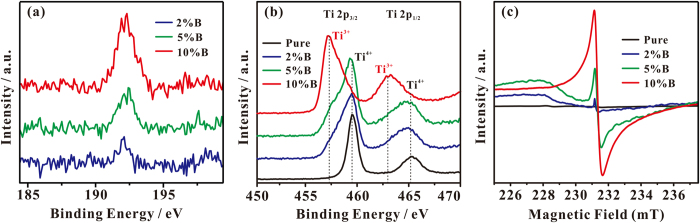
(**a**) B 1 s and (**b**) Ti 2p XPS spectra of B-TiO_2−x_ samples with different B doping. (**c**) ESR spectra of B-TiO_2−x_ samples with different B doping, measured at 295 K.

As shown in Fig. 2a, in the B 1s XPS spectra of the B-TiO_2−x_ samples, the only signal at 191.8 eV is ascribed to the doped B species weaving into the interstitial sites of TiO_2_ lattice (B-O-Ti, called as interstitial B)^7^. Figure 2b shows the Ti 2p XPS spectra of as-prepared pure TiO_2_ and B-TiO_2−x_ photocatalysts. Two major peaks at 459.0 (Ti 2p_3/2_) and 464.7 eV (Ti 2p_1/2_) are observable for pure TiO_2_, being indicative of the sole presence of Ti^4+ ^24,25. Upon introduction of 2% dopant B into TiO_2_, a new Ti 2p_3/2_ XPS shoulder peak appears at 457.5 eV, which can be ascribed to the formation of Ti^3+^ species^26^,^27^. As a surface analysis technique, the detection depth of XPS with the kinetic energy of 40 eV is normally less than 1.0 nm on metallic oxides^28^. Thus, the observed Ti^3+^ species should mainly exist in the surface layer of B-TiO_2−x_. By spectral deconvolution, it was found that the content of Ti^3+^ amounts to ca. 10% of the total surface-layer Ti in the 2% B-TiO_2−x_ photocatalyst. Most interestingly, the Ti 2p_3/2_ XPS signal of Ti^3+^ at 457.5 eV gradually grows up at the expense of the signal of Ti^4+^ at 459.0 eV with the amount of doped B being increased from 2% to 10% in the B-TiO_2−x_ photocatalysts, which unambiguously demonstrates that the additional B doping favors the formation of surface-layer Ti^3+^ species. For the 10% B-TiO_2−x_ photocatalyst, the Ti 2p_1/2_ XPS peak of Ti^3+^ species is clearly observable at 457.3 eV and the content of Ti^3+^ goes up to 87.5% of the total suface-layer Ti. Since Ti^3+^ species is paramagnetic, its existence in B-TiO_2−x_ can be also confirmed by ESR experiments. As shown in Fig. 2c, an ESR signal due to Ti^3+^ with a g-value of 1.987^29^ is observable, and it gradually grows up with the amount of doped B being increased from 2% to 10%, indicating a gradual growth of Ti^3+^. For the 10% B-TiO_2−x_ sample, the concentration of Ti^3+^ species is experimentally estimated to be ca. 30 μmol/g. The high stability of the surface-layer Ti^3+^ species is evidenced by the fact that no obvious change was observable for the ESR and XPS signals after the B-TiO_2−x_ photocatalysts were synthesized and exposed in atmosphere for 18 months.

### Boron-induced structural disorder

**Figure 3:**
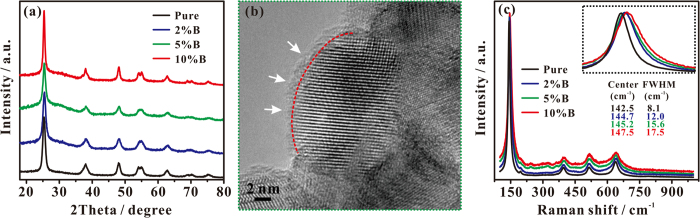
(**a**) XRD spectra of B-TiO_2−x_ samples with different B doping. (**b**) HRTEM image of 10% B-TiO_2−x_. In (**b**), a red dash line is drawn to outline the interface between the crystalline core and the disordered outer layer. (**c**) Raman spectra of pure TiO_2_ and B-TiO_2−x_ samples with different B doping content. Inset: the most intense E_g_ peak at 142.5 cm^−1^, along with its center position and line width (Full Width at Half Maximum, FWHM).

As revealed by X-ray diffraction and high-resolution TEM, the as-synthesized pure TiO_2_ is highly crystallized and exists in form of anatase phase, and the size of individual TiO_2_ nanocrystals is about 21 nm in diameter (see Fig. 3a,b). After the interstitial B doping, the anatase phase remains almost unchanged, while the surface layer of B-TiO_2−x_ becomes disordered (Fig. 3b and Figure S4 in SI), in which the thickness of disordered layer surrounding the crystalline core is ca. 1.5 nm for the 10% B-TiO_2−x_ sample. These structural properties of B-TiO_2−x_ photocatalysts were further examined by Raman scattering spectra (Fig. 3c). For the pure TiO_2_, six Raman-active modes of anatase phase occur with frequencies at ca. 142.5 (E_g_), 196 (E_g_), 396 (B_1g_), 515 (B_1g_ + A_1g_), and 640 (E_g_) cm^−1^, respectively^30^. For the B-TiO_2−x_ photocatalysts, the most intense peak at 142.5 cm^−1^ gradually broadens and blueshifts with the amount of doped B being increased from 2% to 10% (see the inset of Fig. 3c). All these features indicate that notable structural changes occur after the B doping, resulting in the structural disorder in the surface and subsurface layer of B-TiO_2−x_, and the more the B doping the more the structural disorder. Similar conclusion can also be derived from the appearance of new signals at 242.4, 308.2, 358.9, 451.0, and 611.6 cm^−1^. The structural disorders can lead to photon confinement effects, and activate Raman-forbidden modes by breaking down the Raman selection rule, generating the new Raman signals^11^,^31^. Combined with our XPS results, we conclude that sufficient interstitial B doping can generate not only sufficient surface-layer Ti^3+^ species, but also structural disorder in the surface layer of B-TiO_2−x_ photocatalysts.

### Boron environment analysis

**Figure 4:**
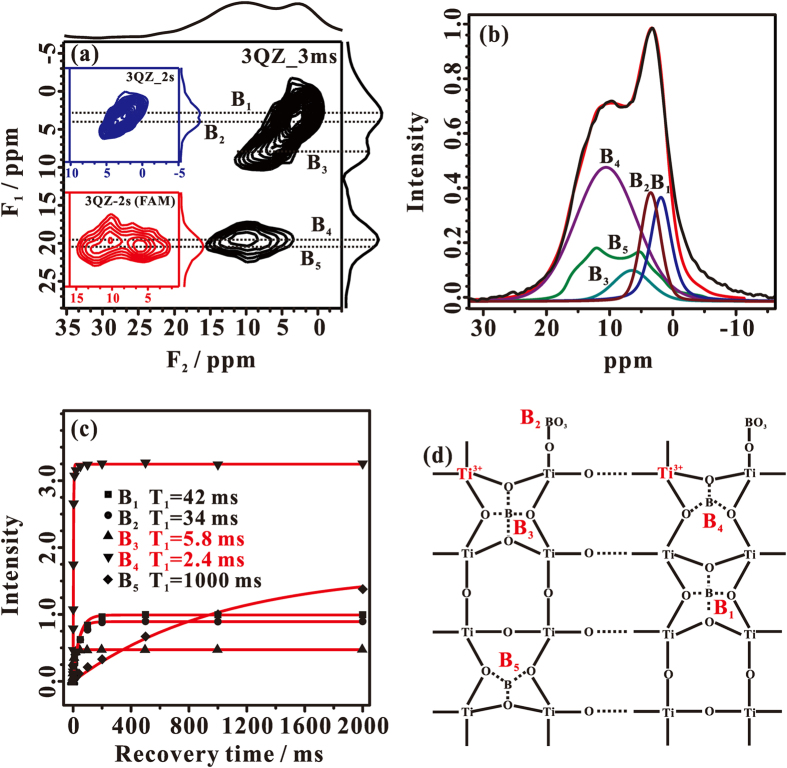
(**a**) The 3QZ MAS NMR spectrum of 10% B-TiO_2−x_ recorded with a extremely short recycle delay of 3.0 ms (3QZ_3 ms). The 3QZ MAS NMR spectrum of 10% B-TiO_2−x_ recorded with an adequate recycle delay of 2 s, is shown in Fig. 4a, insert, upper (3QZ_2 s). To selectively detect the signals with relatively large quadrupolar interactions, the 3QZ-FAM MAS NMR spectrum of 10% B-TiO_2−x_ is recorded with an adequate recycle delay of 2 s and shown in Fig. 4a, insert, down (3QZ_2 s (FAM)). (**b**) Experimental (in black) and simulated (in red) 1D ^11^B MAS spectra together with the deconvoluted spectra corresponding to various B environments. (**c**) Spin-lattice relaxation (T_1_) curves extracted from saturation recovery T_1_ measurements for the different B sites in 10% B-TiO_2−x_. (**d**) Schematic illustration of the five B sites.

The detailed local structure of dopant B in the B-TiO_2−x_ photocatalysts was thoroughly characterized by one-dimensional (1D) and two-dimensional (2D) solid-state ^11^B MAS NMR techniques. When an observed nucleus is in close proximity to a paramagnetic center, its spin-lattice relaxation time (T_1_) would be dramatically reduced. Therefore, we can utilize an extremely short recycle delay (3.0 ms) in the 2D ^11^B triple-quantum z-filtering (3QZ) MAS NMR experiment to emphasize B sites that are in close proximity or bound to the paramagnetic center (surface-layer Ti^3+^). As shown in Fig. 4a, four ^11^B signals (B_1~4_ sites) are resolved in the ^11^B 3QZ MAS NMR spectrum of 10% B-TiO_2−x_. According to the derived isotropic chemical shifts (Table 1), B_1~3_ sites are assigned to tetrahedral-coordinated boron while the B_4_ site is associated with tricoordinated boron^7^. When a long recycle delay of 2 s was used, the B_1_ and B_2_ sites could be much well resolved in the ^11^B 3QZ MAS spectrum (Fig. 4a, insert, upper). In order to detect other possible boron sites with large quadrupolar interactions, we incorporated fast amplitude-modulated (FAM) radiofrequency (RF) pulse trains into the 3QZ MAS pulse sequence, namely the so-called 3QZ-FAM MAS NMR technique^32^, to explore the chemical environments of these boron sites. In addition to the B_4_ signal, a new ^11^B signal (B_5_ site) with a typical second-order quadrupolar line shape appears in the ^11^B 3QZ-FAM MAS NMR spectrum (Fig. 4a, insert, down) acquired with a long recycle delay of 2 s. Therefore, there are five different B sites present in 10% B-TiO_2−x_, which can be utilized for the deconvolution of the 1D ^11^B MAS spectrum (Fig. 4b) to determine the relative content of various B sites (Table 1).

We also measured the spin-lattice relaxation time (T_1_) of the various B sites and the result was shown in Fig. 4c. Except for the B_5_ site, the B_1~4_ sites have extremely short T_1_, indicating that they are in spatial proximity to the surface-layer Ti^3+^ center. Especially, the T_1_ values of B_3_ (2.4 ms) and B_4_ (5.8 ms) sites are even much shorter than those of B_1_ (42 ms) and B_2_ (34 ms) sites, implying that the B_3_ and B_4_ sites may be directly bound to the surface-layer Ti^3+^ center. Since the B_5_ site exhibits a typical T_1_ value (ca. 1000 ms), it should be far away from the surface-layer Ti^3+^ center. According to their isotropic chemical shifts and our previous work^7^, the signals corresponding to B_1_ and B_3_ sites can be assigned to pseudotetrahedral coordinated interstitial boron with the latter being bound to the Ti^3+^ center, while the signal corresponding to B_2_ is due to the surface tetrahedral coordinated boron^7^. Likewise, the signals corresponding to B_4_ and B_5_ sites are assigned to tricoordinated interstitial boron with the former being bound to the surface-layer Ti^3+^ center, and the latter being present in the bulk of B-TiO_2−x_ and far away from the Ti^3+^ center. On the basis of our solid-state ^11^B MAS NMR experimental results, the local chemical environments of different dopant B sites are illustrated in Fig. 4d. The majority of dopant B (B_1_, B_3_, B_4_ and B_5_ species, being ca. 85.4% of the total B) weaves into the interstitial sites of anatase TiO_2_ nanocrystals (Table 1), which not only favors the formation of the Ti^3+^ defects, but also provokes the surface-layer disorders.

### Density of states (DOS) analysis and theoretical calculations

**Figure 5:**
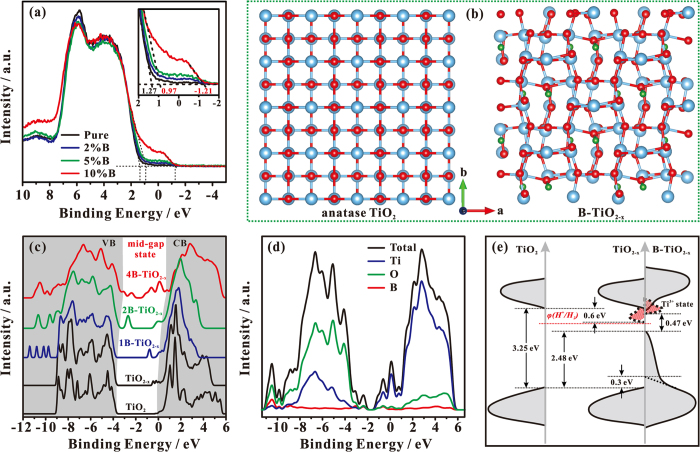
(**a**) Valence-band XPS spectra of various TiO_2_ samples. (**b**) A schematic illustration of supercells of anatase TiO_2_ and 4B-TiO_2−x_ models (red, O atoms; blue, Ti atoms; green, B atoms). (**c**) Calculated DOS of Pure TiO_2_, TiO_2−x_, and various B-TiO_2−x_. (**d**) Decomposition of the total DOS of 4B-TiO_2−x_ into partial DOS of Ti, O, and B orbitals. (**e**) Schematic illustration of the DOS of pure TiO_2_, TiO_2−x_ and B-TiO_2−x_.

The DOSs of the valence band of as-synthesized pure TiO_2_ and B-TiO_2−x_ photocatalysts were measured by valence band XPS technique (Fig. 5a). The pure TiO_2_ displays typical valence band DOS characteristics of TiO_2_, with an edge of maximum energy at ca. 1.27 eV. For the B-TiO_2−x_ samples, a new state was introduced into the band gap with an edge of maximum energy at ca. −1.21 eV. A similar mid-gap state with an edge of maximum energy at ca. −0.92 eV was also found in hydrogenated TiO_2_ nanocrystals, which was due to surface-layer disorders^11^. The intensity of the mid-gap states of B-TiO_2−x_ gradually increases with the amount of doped B being increased. Additionally, the interstitial B doping can lead to a blue-shift for the maximum edge of the original valence band of TiO_2_ (from 1.27 for pure TiO_2_ to 0.97 eV for 10% B-TiO_2−x_), in good agreement with our theoretical results (see below), and the blue-shift increases with the increase of interstitial B doping (Fig. 5a, insert).

In order to determine the structure of active centers and their influence on the electronic structure of the B-TiO_2−x_, we performed theoretical calculations using 2 × 2 × 1 supercell models (Fig. 5b and Figure S5 in SI). It was found that removing a oxygen atom from pure TiO_2_ to form TiO_2−x_ needs to overcome an energy barrier of 132.7 kcal/mol, while this barrier is considerably decreased to 88.9 kcal/mol when a oxygen atom is removed from B-TiO_2_ to form B-TiO_2−x_, which demonstrates that the interstitial B doping favors the generation of Ti^3+^ species in B-TiO_2−x_. To further understand the origin of the change in the electronic and optical properties of B-TiO_2−x_, we also calculated the energy band structures. The schematic illustrations of TiO_2_, TiO_2−x_ and B-TiO_2−x_ supercell models used in the calculations are shown in Fig. 5b and Figure S5 in SI, and the structural parameters of the relaxed structures are listed in Table S1 in SI. It is noteworthy that the supercell models of B-TiO_2−x_ were constructed on the basis of our NMR results. Compared with the standard anatase TiO_2_ supercell, the TiO_2−x_ supercell contains variable O vacancies (associated with Ti^3+^ sites). When one B atom weaves into the interstitial site around an O vacancy, denoted as 1B-TiO_2−x_, the TiO_2−x_ structure is slightly distorted. With further increasing the amount of B and Ti^3+^ in the B-TiO_2−x_ supercell, its lattice becomes more distorted (as for 2B-TiO_2−x_, two O vacancies and two B atoms are present in this case), the symmetry of TiO_2_ lattice is damaged, and eventually the lattice becomes disordered (as for 4B-TiO_2−x_, four O vacancies and four B atoms are present in this case), in consistent with our TEM and Raman experimental observations. These structural changes lead to substantial variations in DOS of B-TiO_2−x_. Figure 5c plots the calculated DOSs of B-TiO_2−x_ along with those of the pure TiO_2_ and TiO_2−x_. Compared with pure TiO_2_, the DOS of TiO_2−x_ shows new electronic states (Ti^3+^ states) below the bottom of the conduction band at 0.8 eV (Fig. 5c), in consistent with the previous report^21^. Although the Ti^3+^ state grows up gradually with the increase of the oxygen vacancy amount, the band gap of TiO_2−x_ remains almost unchanged (see Figure S6 in SI). For the DOS of 1B-TiO_2−x_, a blue-shift (ca. 0.1 eV) is present simultaneously for both valence band and conduction band. In this case, its band gap is similar to that of TiO_2−x_. When more B atoms and O vacancies are gradually doped into the lattice, beside the more blue-shift of valence band and conduction band, two groups of mid-gap states (ranging from ca. −3.8~−2.0 and −1.4~0.5 eV) grows up in the DOS of 2B-TiO_2−x_ and 4B-TiO_2−x_, accompanied by a significant reduction of the band gap. The high-energy mid-gap states (ca. −1.4~0.5 eV) can be mainly ascribed to the formation of Ti^3+^ (O vacancy) states, while the low-energy mid-gap states (ca. −3.8~−2.0 eV) are mainly due to the lattice disorder. The project DOSs of B-TiO_2−x_ are shown in Fig. 5d and Figure S6 in SI. It can be seen that the two groups of mid-gap states are hybridized with Ti, O, and B states. Therefore, the B atoms coupling to the neighboring lattice O and Ti atoms in the interstitial or defect sites of B-TiO_2−x_ do make a substantial contribution to the two mid-gap states, suggesting that the B doping can favor the formation and stabilization of the Ti^3+^ species as well as the lattice disorder.

### Understanding the high catalytic activity of B-TiO_2−x_

Based on our experimental and theoretical results, a schematic illustration of the DOS of B-TiO_2−x_ is proposed in Fig. 5e. The band gap of pure TiO_2_ is ca. 3.25 eV. According to our theoretical calculations, the existence of abundant Ti^3+^ species induces an electronic state just below the conduction band minimum as shown in Fig. 5c and Figure S6 in SI, in consistent with the previous reports^21^. However, when the amount of Ti^3+^ is high enough (i.e. in the TiO_2−x_ (4Vo) sample), more Ti^3+^ states can be introduced in the band gap of DOS as shown in Figure S5, which may prevent the improvement of photocatalytic H_2_ production due to the electric potential of some Ti^3+^ states being less than that of H^+^ reduction (*φ(H*^+^*/H*_*2*_)). When the additional interstitial B is doped, a blue-shift is simultaneously present for valence band and conduction band in the DOSs of B-TiO_2−x_ compared with the corresponding DOSs of TiO_2−x_. Furthermore, when an appropriate amount of interstitial B (in the 2B-TiO_2−x_) is doped arround the Ti^3+^ site, the blue-shift is also present for the Ti^3+^ states (Figure S6 in SI). The blue-shift of both conduction band and Ti^3+^ states can result in a higher electric potential than that of TiO_2−x_ with respect to *φ*(*H*^+^/*H*_*2*_), and thus easier H_2_ production. It has been validated experimentally and theoretically that the sufficient interstitial B doping not only favors the formation of abundant Ti^3+^ defects, but also provokes the surface-layer disorder. According to the valence band XPS spectra and theoretical calculations (Fig. 5), the surface-layer disorder causes the valence band maximum energy blue-shift by ca. 2.5 eV toward the vacuum level. Thus, a much narrowed band gap of ca. 0.47 eV is present in the DOS of B-TiO_2−x_, in consistent with our optical measurement result (<0.92 eV). As such, it can be concluded that the visible-light absorption of B-TiO_2−x_ would be much better than those of both TiO_2−x_ and pure TiO_2_. More interestingly, the structural changes (including the formation of B-O-Ti^3+^ bond, the resultant structural distortion, and the surface disorder) can result in the formation of band tail state (Fig. 5). As a result, a better overlapping between the mid-gap states and the original energy band states of TiO_2_ is present in the B-TiO_2−x_ photocatalysts as shown in Fig. 5e. Therefore, the band gap engineering can promote the mobility of photogenerated carriers (electron and hole), while the corresponding two mid-gap states, provoked by surface disorder and B-O-Ti^3+^ structure, may trap shallowly the photogenerated carriers to prevent them from rapid recombination, which eventually improves the solar-light photocatalytic activity of B-TiO_2−x_.

## Conclusion

In summary, we have developed a novel and simple approach to generate and stabilize the sufficient Ti^3+^ species in the surface layer of TiO_2_ by introducing interstitial B. Our experimental and computational results unambiguously demonstrate that the introduction of interstitial B not only leads to the formation of abundant and stable Ti^3+^ species but also generates structural disorder in the surface layer of B-TiO_2−x_ photocatalysts. These structural changes lead to substantial variations in their density of states, that is, two new groups of mid-gap states are formed, accompanied by a significant reduction of the band gap. These band gap engineering not only can promote the photogenerated electron mobility but also provide shallow trapping sites (B-O-Ti^3+^) for photogenerated carriers to prevent them from rapid recombination, which eventually improves the solar-light photocatalytic activity of B-TiO_2−x_ toward hydrogen production water splitting. The experimental and theoretical calculation results presented herein should not only facilitate a better understanding of the photocatalytic mechanism at the atomic level but also be helpful for rational design of highly efficient titania-based photocatalysts.

## Methods

### Synthesis

A series of B-TiO_2−x_ photocatalysts were prepared with a precursor solution consisting of titanium tetrachloride, boric acid, ethanol, and deionized water, with molar ratios of TiCl_4_/H_3_BO_3_/NaCl/H_2_O/ethanol at 1 : × : 2 : 60 : 20 (x = 0, 0.02, 0.05, and 0.10). The solution was stirred for ca. 2.5 days in ice bath, and then its pH value was adjusted to 7.0 by ammonia. The mixture was aged for 24 h at room temperature, washed with water to remove NH_4_Cl and NaCl during the filtration, and then dried at 80 °C. After being calcined at 450 °C in N_2_/air for 5 h, the resultant grey and blue powder was well-crystallized with features assignable to anatase crystalline phase as determined by X-ray diffraction. The actual B doping amount (molar ratio, B/Ti) is deterimned by solid-state NMR analysis, and it is ca. 1.6%, 3.9%, and 7.2% in 2% B-TiO_2−x_, 5% B-TiO_2−x_, and 10% B-TiO_2−x_, respectively.

### Photocatalytic activity

After loading a sample (0.005 g) with 0.6% Pt (0.00003 g), the photocatalyst (Pt/B-TiO_2−x_) was placed into an aqueous methanol solution (5.0 ml, 5.0 volume %. Concerning Pt deposition, 0.2 g of the as-prepared TiO_2_ photocatalysts were dispersed in a 10 mL aqueous solution (0.618 mM H_2_PtCl_6_•6H_2_O) with stirring, which was irradiated using a 300 W Hg lamp for 2 h. Methanol acts as the sacrificial reagent in a Quartz glass container. The simulated solar light by Xe lamp (300 W) was used as the excitation source, which produced a power of ca. 130 mW/cm^2^. The hydrogen gas in reactor was replaced with nitrogen for 30 min before each cycling test. The energy conversion efficiency was calculated based on the equation 2H/P. Here H was the number of moles of hydrogen gas produced during a specific time, while P was the number of photons incident on the photocatalytic system within the same time period.

### Characterization Methods

The crystalline structures of various photocatalysts were determined by X-ray diffraction (XRD) on a Bruker D/max2550 instrument using Cu KR radiation (40 kV, 40 kmA). All UV-Vis diffuse reflectance spectroscopic (DRS) studies were carried out on an Agilent Technologies Cary 4000/5000 spectrophotometer using BaSO_4_ as the reference. The high resolution transmission electron microscope (HRTEM) images were taken using a JEOL 2010 electron microscope operating at 200 kV. Samples of each catalyst were prepared for TEM by dispersing the photocatalyst powder in high-purity ethanol. A drop of the suspension was then allowed to evaporate on a holey-carbon film supported by a 300-mesh copper TEM grid.

X-ray photoelectron spectroscopy (XPS) spectra were recorded on a Kratos Axis Ultra delay line detector (DLD) spectrometer equipped with a monochromatic Al KR X-ray source (hʋ = 1486.6 eV), hybrid (magnetic/electrostatic) optics with a multichannel plate, and DLD. All XPS spectra were recorded using an aperture slot of 300 × 700 μm. Survey spectra were recorded with an energy of 160 eV, as compared to the high resolution spectra (40 eV). The accuracy of the XPS binding energies (BE) is 0.1 eV.

ESR measurements were carried on a JEOL JES-FA200 ESR spectrometer at room temperature. The TiO_2_ photocatalysts were transfered into ESR tube in the air atmosphere, followed by degassing (to remove gaseous O_2_) at room temperature on a vacuum line. TEMPO (2, 2, 6, 6 - Tetramethyl - 1 - piperidinyloxy) was used as an external reference for quantification of Ti^3+^ concentration.

All solid-state nuclear magnetic resonance (NMR) experiments were performed on a Varian Infinitypuls-400 spectrometer using a Chemagnetic 4 mm double resonance probe. A Larmor frequency of 128.38 MHz, and a typical π/2 pulse length of 2.5 μs were adopted for ^11^B resonance, respectively. The excitation pulse length was adjusted to π/12 for the single-pulse ^11^B MAS experiments, in which a repetition time of 2 s and a total of 20000 accumulated scans were used. The ^11^B 3QZ MAS and 3QZ-FAM MAS NMR spectra were recorded with the pulse sequence proposed by Vega *et al*.^32^, in which 64 t1 increments of 10 μs in the F1 dimension were acquired (collecting 2880 scans per t1 increment) with a recycle delay of 3.0 ms or 2.0 s under a sample spinning rate of 15.0 kHz. All ^11^B NMR chemical shifts were referenced to that of H_3_BO_3_ (0.1 M).

### Computational Methods

Theoretical calculations were based on density functional theory (DFT) and performed by using VASP simulation package^33^. The exchange and correlation functionals with a Perdew-Burke-Ernzerhof generalized gradient corrected approximation (GGA)^34^ and projector-augmented wave (PAW)^35^ pseudopotentials describing the electron–ion interactions were used in the calculations. The wave function was described with a plane wave basis set and an energy cutoff of 400 eV was used. The *k*-point meshes employed in the calculations were generated according to the Monkhorst-Pack scheme^36^. The resulting Brillouin-Zone sampling used for supercells was equivalent to the one obtained with (10 × 10 × 3) grids for the pristine anatase-TiO_2_ bulk. Good convergence was achieved with this cutoff energy and the number of *k* points for the various structures considered. B-doped TiO_2_ with variable O vacancies (denoted as B-TiO_2−x_) were modeled by (2 × 2 × 1) supercell of crystal TiO_2_. Several cases with different B concentrations (from 1B to 4B) were considered in the calculations. For a certain B concentration, several possible initial configurations were optimized and the electronic structures were calculated for the most stable configurations and meta-stable configurations. The relaxed structures for most stable configurations of B-TiO_2−x_, together with TiO_2−x_ were illustrated. The Heyd, Scuseria and Ernzerhof (HSE) hybrid functional^37^ has been used to calculate DOS of pure TiO_2_, TiO_2−x_, and B-TiO_2−x_.

Additional experimental and theoretical calculation details can be found in the supporting information.

## Additional Information

**How to cite this article**: Feng, N. *et al*. Unravelling the Efficient Photocatalytic Activity of Boron-induced Ti^3+^ Species in the Surface Layer of TiO_2_. *Sci. Rep*. **6**, 34765; doi: 10.1038/srep34765 (2016).

## Supplementary Material

Supplementary Information

## Figures and Tables

**Table 1:** NMR Parameters Derived from ^11^B NMR Results for Various Boron Species in B-TiO_2−x_.

Site	δ_iso_ (ppm)	QCC^b^/PQ^a^ (MHz)	η	δ_MAS_ (ppm) F_2_	δ_MAS_ (ppm) F_1_	Content %
B_1_	2.6	0.46^a^	—	2.4	2.9	14.1
B_2_	4.0	0.29^a^	—	3.8	4.0	14.6
B_3_	7.9	0.71^a^	—	6.8	8.0	8.5
B_4_	16.3	2.02^a^	—	10.8	19.5	49.8
B_5_	16.3	2.23^b^	0.30	9.0	20.5	13.0

^a^Derived from eq. S2.

^b^Extracted by spectral fitting using the Dmfit program.

## References

[b1] Fujishima A. & Honda K. Electrochemical Photolysis of Water at a Semiconductor Electrode. Nature 238, 37–38 (1972). 12635268 10.1038/238037a0

[b2] Ghosh A. K. & Maruska H. P. Photoelectrolysis of Water in Sunlight with Sensitized Semiconductor Electrodes. J. Electrochem. Soc. 124, 1516–1522 (1977).

[b3] Choi W., Termin A. & Hoffmann M. R. Effects of Metal-Ion Dopants on the Photocatalytic Reactivity of Quantum-Sized TiO_2_ Particles. Angew. Chem. Int. Ed. 33, 1091–1092 (1994).

[b4] Choi W., Termin A. & Hoffmann M. R. The Role of Metal Ion Dopants in Quantum-Sized TiO_2_: Correlation between Photoreactivity and Charge Carrier Recombination Dynamics. J. Phys. Chem. 98, 13669–13679 (1994).

[b5] Asahi R., Morikawa T., Ohwaki T., Aoki K. & Taga Y. Visible-Light Photocatalysis in Nitrogen-Doped Titanium Oxides. Science 293, 269–271 (2001). 11452117 10.1126/science.1061051

[b6] Khan S. U. M., Al-Shahry M. & Ingler W. B. Jr. Efficient Photochemical Water Splitting by a Chemically Modified n-TiO_2_. Science 297, 2243–2245 (2002). 12351783 10.1126/science.1075035

[b7] Feng N. . Boron Environments in B-Doped and (B, N)-Codoped TiO_2_ Photocatalysts: A Combined Solid-State NMR and Theoretical Calculation Study. J. Phys. Chem. C 115, 2709–2719 (2011).

[b8] Liu G. . A red anatase TiO_2_ photocatalyst for solar energy conversion. Energy Environ. Sci. 5, 9603–9610 (2012).

[b9] Sun C. & Searles D. J. Origin of the Visible Light Absorption of Boron/Nitrogen Co-doped Anatase TiO_2_. J. Phys. Chem. C 117, 26454–26459 (2013).

[b10] Anpo M. Photocatalysis on titanium oxide catalysts Approaches in achieving highly efficient reactions and realizing the use of visible light. Catal. Surv. Jpn. 1, 169–179 (1997).

[b11] Chen X., Liu L., Yu P. Y. & Mao S. S. Increasing Solar Absorption for Photocatalysis with Black Hydrogenated Titanium Dioxide Nanocrystals. Science 331, 746–750 (2011). 21252313 10.1126/science.1200448

[b12] Sirisuk A., Klansorn E. & Praserthdam P. Effects of reaction medium and crystallite size on Ti^3+^ surface defects in titanium dioxide nanoparticles prepared by solvothermal method. Catal. Commun. 9, 1810–1814 (2008).

[b13] Zuo F. . Active Facets on Titanium(III)-Doped TiO_2_: An Effective Strategy to Improve the Visible-Light Photocatalytic Activity. Angew. Chem. Int. Ed. 51, 6223–6226 (2012). 10.1002/anie.201202191 22566101

[b14] Zuo F. . Self-Doped Ti^3+^ Enhanced Photocatalyst for Hydrogen Production under Visible Light. J. Am. Chem. Soc. 132, 11856–11857 (2010). 20687606 10.1021/ja103843d

[b15] Hoang S., Berglund S. P., Hahn N. T., Bard A. J. & Mullins C. B. Enhancing Visible Light Photo-oxidation of Water with TiO_2_ Nanowire Arrays via Cotreatment with H_2_ and NH_3_: Synergistic Effects between Ti^3+^ and N. J. Am. Chem. Soc. 134, 3659–3662 (2012). 22316385 10.1021/ja211369s

[b16] Xing M. . Self-doped Ti^3+^-enhanced TiO_2_ nanoparticles with a high- performance photocatalysis. J. Catal. 297, 236–243 (2013).

[b17] Xing M., Li X. & Zhang J. Synergistic effect on the visible light activity of Ti^3+^ doped TiO_2_ nanorods/boron doped graphene composite. Sci. Rep. 4, 5493 (2014). 24974890 10.1038/srep05493 PMC4074785

[b18] Xing M., Fang W., Yang X., Tian B. & Zhang J. Highly-dispersed boron-doped graphene nanoribbons with enhanced conductibility and photocatalysis. Chem. Commun. 50, 6637–6640 (2014). 10.1039/c4cc01341g 24825321

[b19] Czoska A. M. . The nitrogen–boron paramagnetic center in visible light sensitized N–B co-doped TiO_2_. Experimental and theoretical characterization. Phys. Chem. Chem. Phys. 13, 136–143 (2011). 21038048 10.1039/c0cp00143k

[b20] Cronemeyer D. C. Infrared Absorption of Reduced Rutile TiO_2_ Single Crystals. Phys. Rev. 113, 1222–1226 (1959).

[b21] Justicia I. . Designed self-doped titanium oxide thin films for efficient visible-light photocatalysis. Adv. Mater. 14, 1399–1402 (2002).

[b22] Thompson T. L. & Yates J. T. Surface Science Studies of the Photoactivation of TiO_2_-New Photochemical Processes. Chem. Rev. 106, 4428–4453 (2006). 17031993 10.1021/cr050172k

[b23] Teleki A. & Pratsinis S. E. Blue nano titania made in diffusion flames. Phys. Chem. Chem. Phys. 11, 3742–3747 (2009). 19421486 10.1039/b821590a

[b24] Liu M., Qiu X., Miyauchi M. & Hashimoto K. Cu(II) Oxide Amorphous Nanoclusters Grafted Ti^3+^ Self-Doped TiO_2_: An Efficient Visible Light Photocatalyst. Chem. Mater. 23, 5282–5286 (2011).

[b25] Lu X. . Hydrogenated TiO_2_ Nanotube Arrays for Supercapacitors. Nano Lett. 12, 1690–1696 (2012). 22364294 10.1021/nl300173j

[b26] Werfel F. & Brummer O. Corundum Structure Oxides Studied by XPS. Phys. Scr. 28, 92–96 (1983).

[b27] McCafferty E. & Wightman J. P. Determination of the concentration of surface hydroxyl groups on metal oxide films by a quantitative XPS method. Surf. Interface Anal. 26, 549–564 (1998).

[b28] Kato S. . Quantitative depth profiling of Ce^3+^ in Pt/CeO_2_ by *in situ* high-energy XPS in a hydrogen atmosphere. Phys. Chem. Chem. Phys. 17, 5078–5083 (2015). 25599521 10.1039/c4cp05643d

[b29] Conesa J. C. & Soria J. Reversible titanium(3+) formation by hydrogen adsorption on M/anatase (TiO_2_) catalysts. J. Phys. Chem. 86, 1392–1395 (1982).

[b30] Chen X. & Mao S. S. Titanium Dioxide Nanomaterials: Synthesis, Properties, Modifications, and Applications. Chem. Rev. 107, 2891–2959 (2007). 17590053 10.1021/cr0500535

[b31] Yu P. Y. & Cardona M. Fundamentals of Semiconductors: Physics and Materials Properties (Springer, Heidelberg, ed. 4, 2010).

[b32] Madhu P. K., Goldbourt A., Frydman L. & Vega S. Sensitivity enhancement of the MQMAS NMR experiment by fast amplitude modulation of the pulses. Chem. Phys. Lett. 307, 41–47 (1999).

[b33] Kresse G. & Furthmuller J. Efficient iterative schemes for ab initio total-energy calculations using a plane-wave basis set. Phys. Rev. B 54, 11169–11186 (1996). 10.1103/physrevb.54.11169 9984901

[b34] Perdew J. P., Burke K. & Ernzerhof M. Generalized Gradient Approximation Made Simple. Phys. Rev. Lett. 77, 3865–3868 (1996). 10062328 10.1103/PhysRevLett.77.3865

[b35] Kresse G. & Joubert D. From ultrasoft pseudopotentials to the projector augmented-wave method. Phys. Rev. B 59, 1758–1775 (1999).

[b36] Monkhorst H. J. & Pack J. D. Special points for Brillouin-zone integrations. Phys. Rev. B 13, 5188–5192 (1976).

[b37] Heyd J., Scuseria G. E. & Ernzerhof M. Hybrid functionals based on a screened Coulomb potential. J. Chem. Phys. 118, 8207–8215 (2003).

