# Supplementary material for: Unravelling the Efficient Photocatalytic Activity of Boron-induced Ti3+ Species in the Surface Layer of TiO2
Source: Sci Rep. 2016 Oct 6;6:34765. doi: 10.1038/srep34765 (PMC5052528; doi:10.1038/srep34765)
Supplement: Supplementary Information [file srep34765-s1.doc]

***Supplementary Information***

**Unravelling the Efficient Photocatalytic Activity of Boron-induced Ti^3+^ Species in the Surface Layer of TiO_2_**

Ningdong Feng^a, †^, Fen Liu^a, †^, Min Huang^a^, Anmin Zheng^a^, Qiang Wang^a^, Tiehong Chen^c^, Gengyu Cao^a^, Jun Xu^a^, Jie Fan^b^, Feng Deng^a^, *

^a^ State Key Laboratory of Magnetic Resonance and Atomic and Molecular Physics, National Center for Magnetic Resonance in Wuhan, Wuhan Institute of Physics and Mathematics, Chinese Academy of Sciences, Wuhan 430071, China.

^b^ Key Laboratory of Applied Chemistry of Zhejiang Province and Department of Chemistry, Zhejiang University, Hangzhou 310027, China.

*^c^* *Key Laboratory of Functional Polymer Materials of MOE, Department of Materials Chemistry, Nankai University, Tianjin 300071, P.R. China.*

* To whom correspondence should be addressed. E-mail: [dengf@wipm.ac.cn](mailto:dengf@wipm.ac.cn) Fax: +86-27-87199291, ^†^ Both authors contributed equally to this work.

**Calculation of NMR parameters.** The isotropic chemical shift (δ_iso_) and quadrupolar interaction parameter (P_Q_) were estimated by the center of gravity of the signal corresponding to the F1 and F2 axes (designated by δ_F1_ and δ_F2_) observed in the 3QZ MAS NMR spectra (sheared) in Figure 4 (see main text) using the following equations:

$\delta_{\text{iso}}\text{ }=\text{ }\frac{17}{27}\delta_{\text{F1}}\text{ }+\text{ }\frac{10}{27}\delta_{\text{F2}}$; Eq. (S1)

$\text{P}_{\text{Q}}\text{ }=\text{ }\upsilon_{0}\times\sqrt{\frac{17}{675000}\times\text{(}\delta_{\text{F1}}\text{- }\delta_{\text{F2}}\text{)}}\text{ = }Q\text{CC}\times\sqrt{\text{1+}\frac{\eta^{2}}{3}}$, Eq. (S2)

where υ_0_ denote the Larmor frequency. Accordingly, the NMR parameters, *viz.* isotropic chemical shift (δ_iso_), quadrupolar coupling constant (QCC), and asymmetry parameter (η) corresponding to various B sites may be extracted by fitting of the second-order quadrupolar line shape obtained from slices of the ^11^B 3QZ-FAM MAS NMR spectra (Figure 4a) using the Dmfit program ^1^. In turn, these NMR parameters were adopted to simulate the 1D NMR spectra in Figure 4b.

The NMR parameters of the B_5_ site (Table 1 in main text) were derived from fitting of the second-order quadrupolar line shape obtained from slices of the ^11^B 3QZ-FAM MAS NMR spectra (Figure 4a, insert, down).

**Theoretical Calculations.** Theoretical calculations were based on density functional theory (DFT) and have been performed by using VASP simulation package ^2^. The exchange and correlation functionals with a Perdew-Burke-Ernzerhof generalized gradient corrected approximation (GGA) ^3^ and projector-augmented wave (PAW) ^4^ pseudopotentials describing the electron–ion interactions were used in the calculations. The wave function was described with a plane wave basis set and an energy cutoff of 400 eV was used. The *k*-point meshes employed in the calculations were generated according to the Monkhorst-Pack scheme ^5^. The resulting Brillouin-Zone sampling used for supercells was equivalent to the one obtained with (10×10×3) grids for the pristine anatase-TiO_2_ bulk. Good convergence was achieved with this cutoff energy and the number of *k* points for the various structures considered. The obtained lattice constant for anatase-TiO_2_ crystal is *a=b*=3.781Å, which is in good agreement with the experimental values ^6^. The lattice constant *c* was calculated to be 9.78 Å, which is slightly larger than the experimental values (9.50 Å) ^6^, however consistent with previous DFT calculations ^7^. The experimental values of the lattice constants (*a, b* and *c*) of anatase-TiO_2_ were used in our calculations.

B-doped TiO_2_ with O vacancies (denoted as B-TiO_2-x_) were modeled by (2×2×1) supercell of crystal TiO_2_. Several cases with different B concentrations (from 1B to 4B) were considered in the calculations. For a certain B concentration, several possible initial configurations were optimized and the electronic structures were calculated for the most stable configurations and meta-stable configurations. The relaxed structures for most stable configurations of B-TiO_2-x_, together with TiO_2-x_ are shown in Figure S5. It was found that the higher B concentration doped in TiO_2_, the larger distortion of the structures of B-TiO_2-x_. The structural parameters for the most stable configurations of B doped TiO_2-x_ are shown Table S1. It is clear from Table S1 that not only the lattice constant (*a, b* and *c*) but also the shapes of the supercells (*α, β* and *γ*) were distorted upon the B doping. For the case of 1B-TiO_2-x_ with the lowest B concentration, the distortions mainly located near B sites due to the formation of new B-O bonds. The supercell for 4B-TiO_2-x_, which has the highest B concentration among the structures possessed the largest distortion. Such distortions in structures may result in the different electronic structures of the B-doped TiO_2-x_.

It is well-know that standard DFT underestimates the band gap of semiconductors to some extent. Therefore, we compared the total density of states (DOS) bulk TiO_2_ calculated by standard DFT and Heyd, Scuseria and Ernzerhof (HSE) hybrid functional ^8^ as shown in the following Figure. It is clear that the calculated band gap for bulk TiO_2_ is around 2.0 eV for the standard DFT calculation and 3.30 eV for the HSE calculation. The HSE value is in good agreement with the experimental result (3.3 eV). In order to better understand the electronic states of Ti^3+^, the DOS of TiO_2-x_ and B-TiO_2-x_ were calculated by HSE for their most stable structures relaxed by standard DFT.

Figure of calculated DOS for bulk TiO_2_ calculated by both standard DFT and HSE.

The calculated HSE total DOS and partial DOS of bulk TiO_2_, TiO_2-x_ and various B-TiO_2-x_ considered were shown in Figure S6. It is clear that the broadened tails of states at both valance band maximum (VBM) and CBM were induced upon the doping of B in TiO_2_.

**Table S1.** Structural parameters of the relaxed structures of bulk TiO_2_, TiO_2-x_ and B-TiO_2-x_.

|  | *a* (Å) | *b* (Å) | *c* (Å) | *α* (^o^) | *β* (^o^) | *γ* (^o^) |
| --- | --- | --- | --- | --- | --- | --- |
| TiO_2_ | 7.552 | 7.552 | 9.486 | 90.000 | 90.000 | 90.000 |
| TiO_2-x_ | 7.552 | 7.552 | 9.486 | 90.000 | 90.000 | 90.000 |
| 1B-TiO_2-x_ | 7.598 | 7.654 | 9.427 | 90.061 | 90.000 | 90.000 |
| 2B-TiO_2-x_ | 7.532 | 7.617 | 9.564 | 90.000 | 90.000 | 90.139 |
| 4B-TiO_2-x_ | 7.755 | 7.639 | 9.160 | 89.994 | 89.986 | 90.080 |


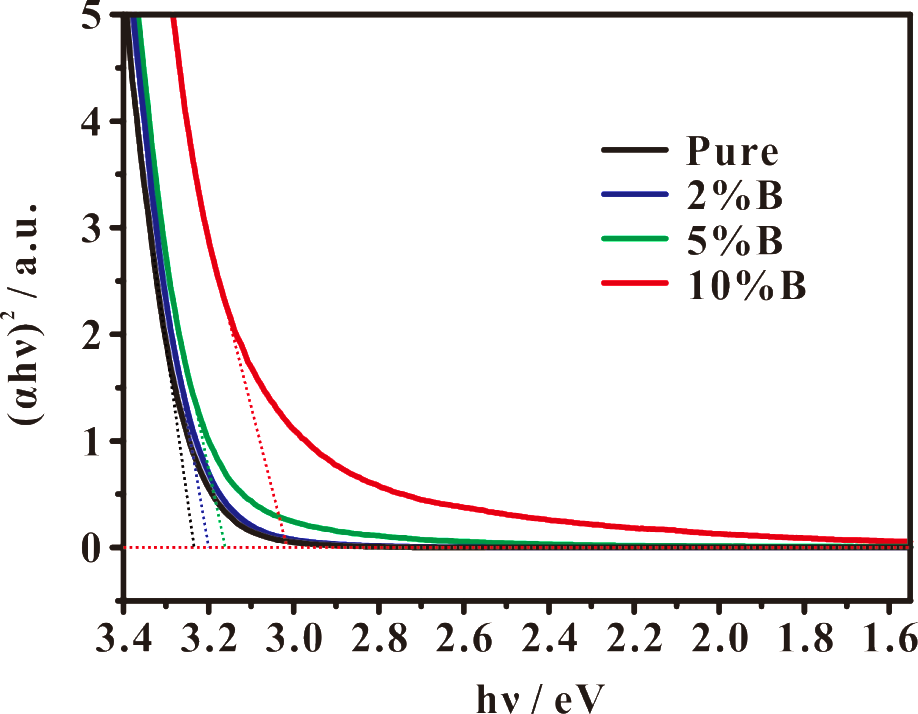


**Figure S1.** Plot of the transformed Kubelka-Munk function against photo energy to the UV-Vis spectra of pure TiO_2_ and B-TiO_2-x_ samples with various B-doping content.


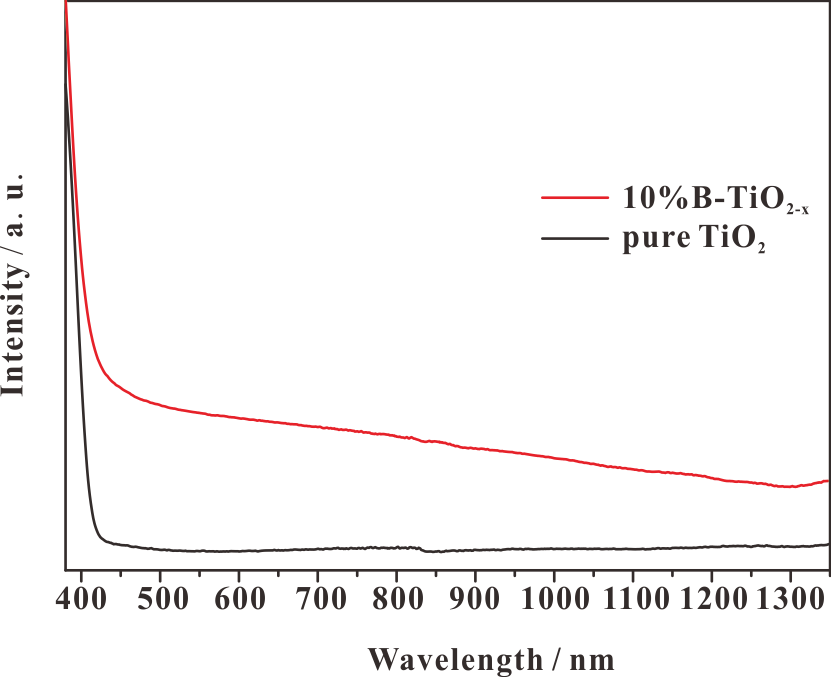


**Figure S2.** UV−Vis absorption spectra of pure TiO_2_ and 10% B-TiO_2-x_ samples.


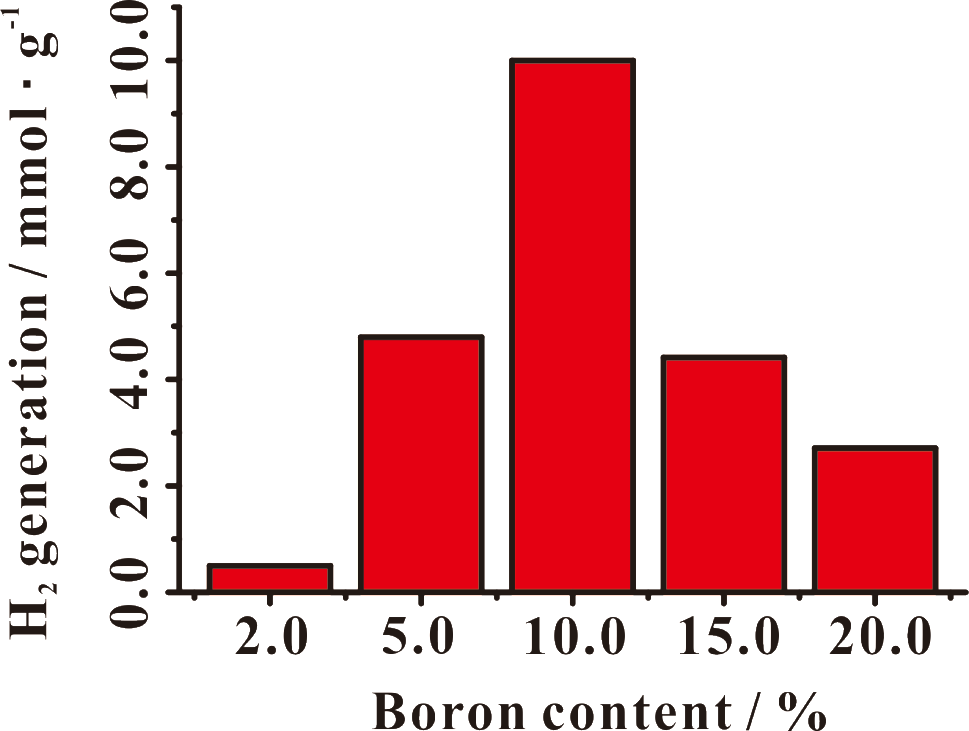


**Figure S3.** The photocatalytic activity of B-TiO_2-x_ with different B content in water splitting hydrogen production under simulated solar-light irradiation


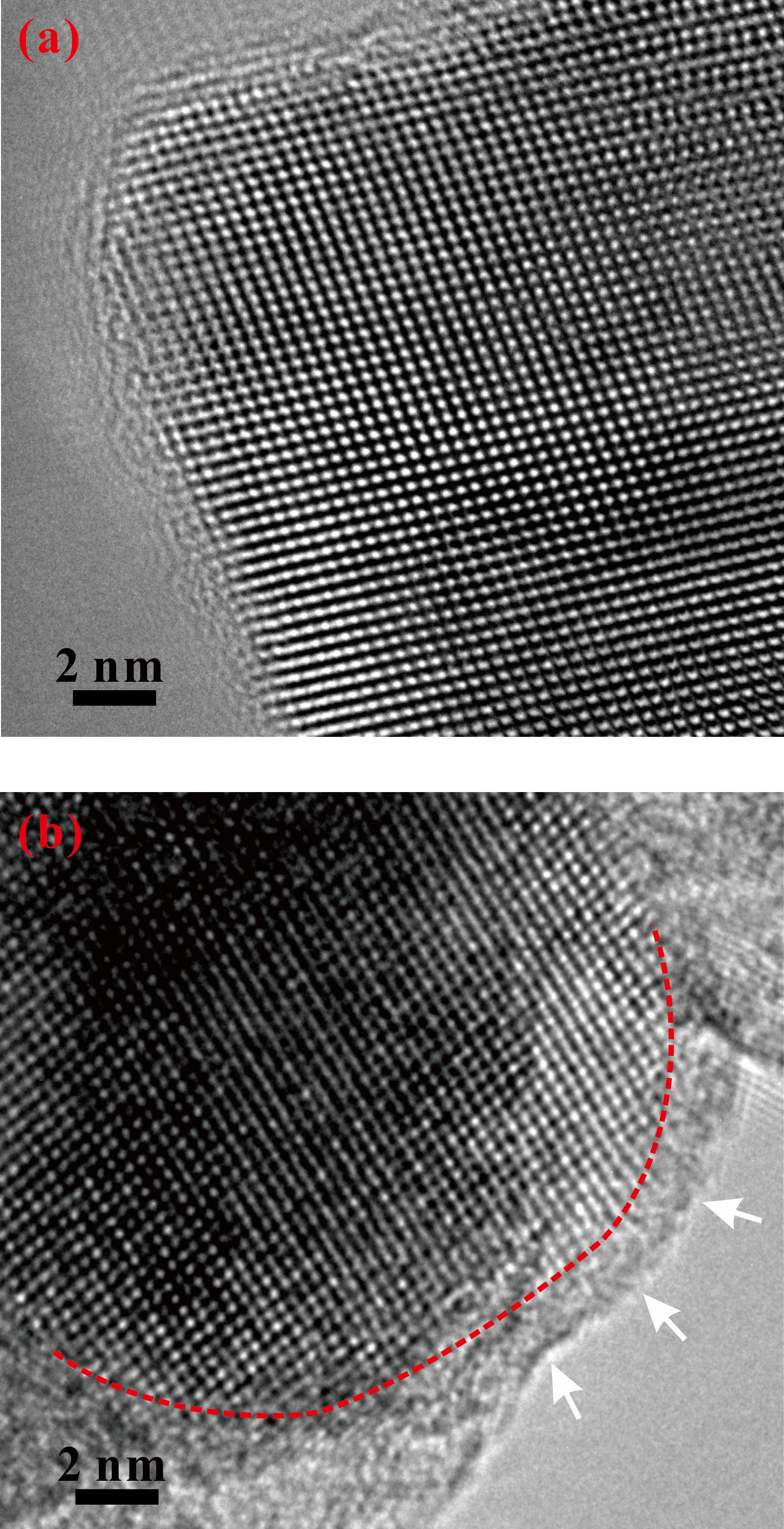


**Figure S4.** HRTEM images of (a) pure TiO_2_ and (b) 10% B-TiO_2-x_. A red dash line is drawn to outline the interface between crystalline core and disordered outer layer of 10% B-TiO_2-x_. The HRTEM image of another selected 10% B-TiO_2-x_ nanoparticle having different crystal plane with respective to that shown in Figure 3b is shown in Figure S3b.


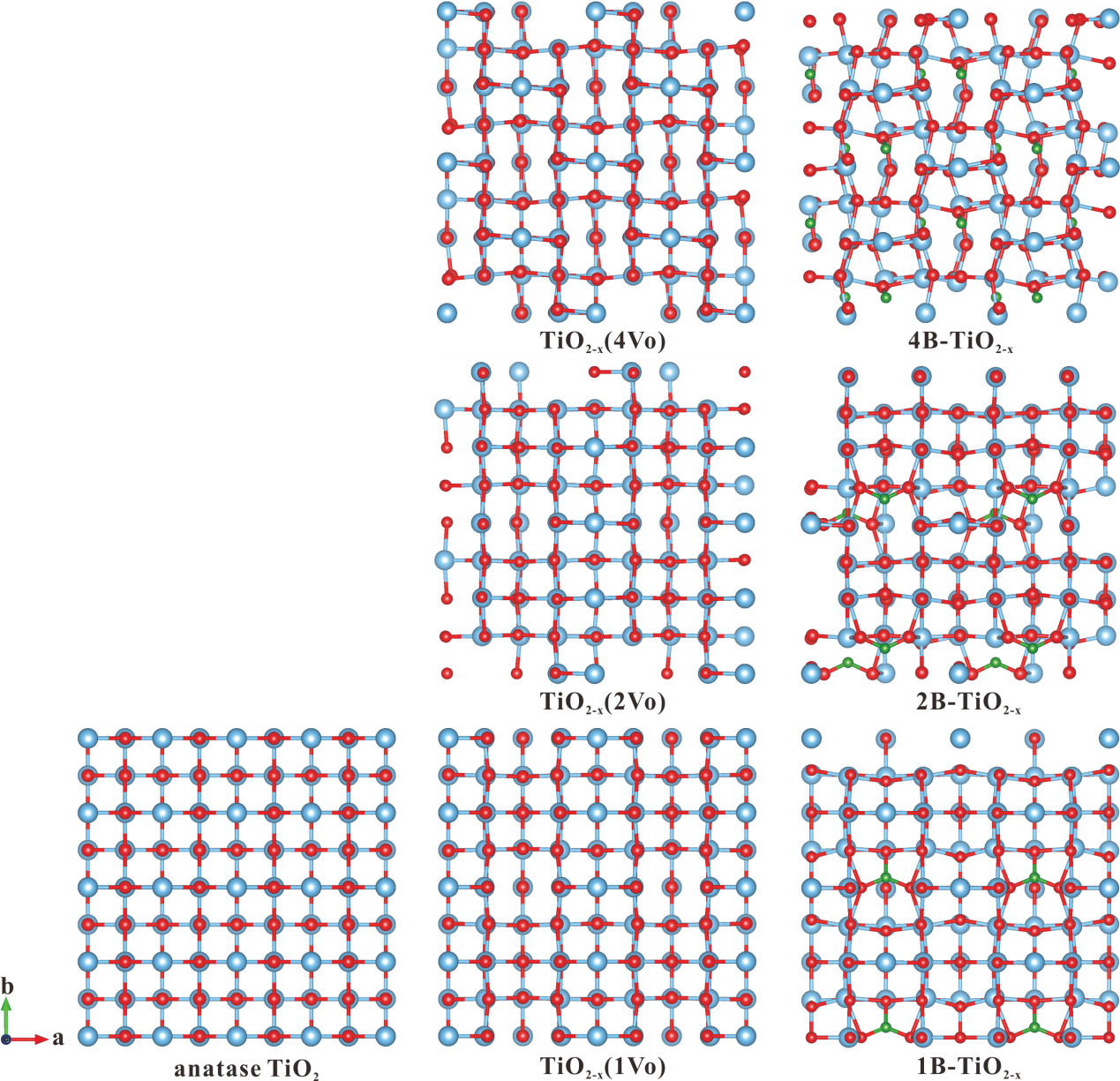


**Figure S5.** Schematic illustration of a supercell for modeling TiO_2_, various TiO_2-x_ with variable oxygen vacancy (Vo) and various B-TiO_2-x_ with variable B doping (red, O atoms; blue, Ti atoms; green, B atoms). To illustrate better, all the supercells were expanded two times along both *a* and *b* directions.


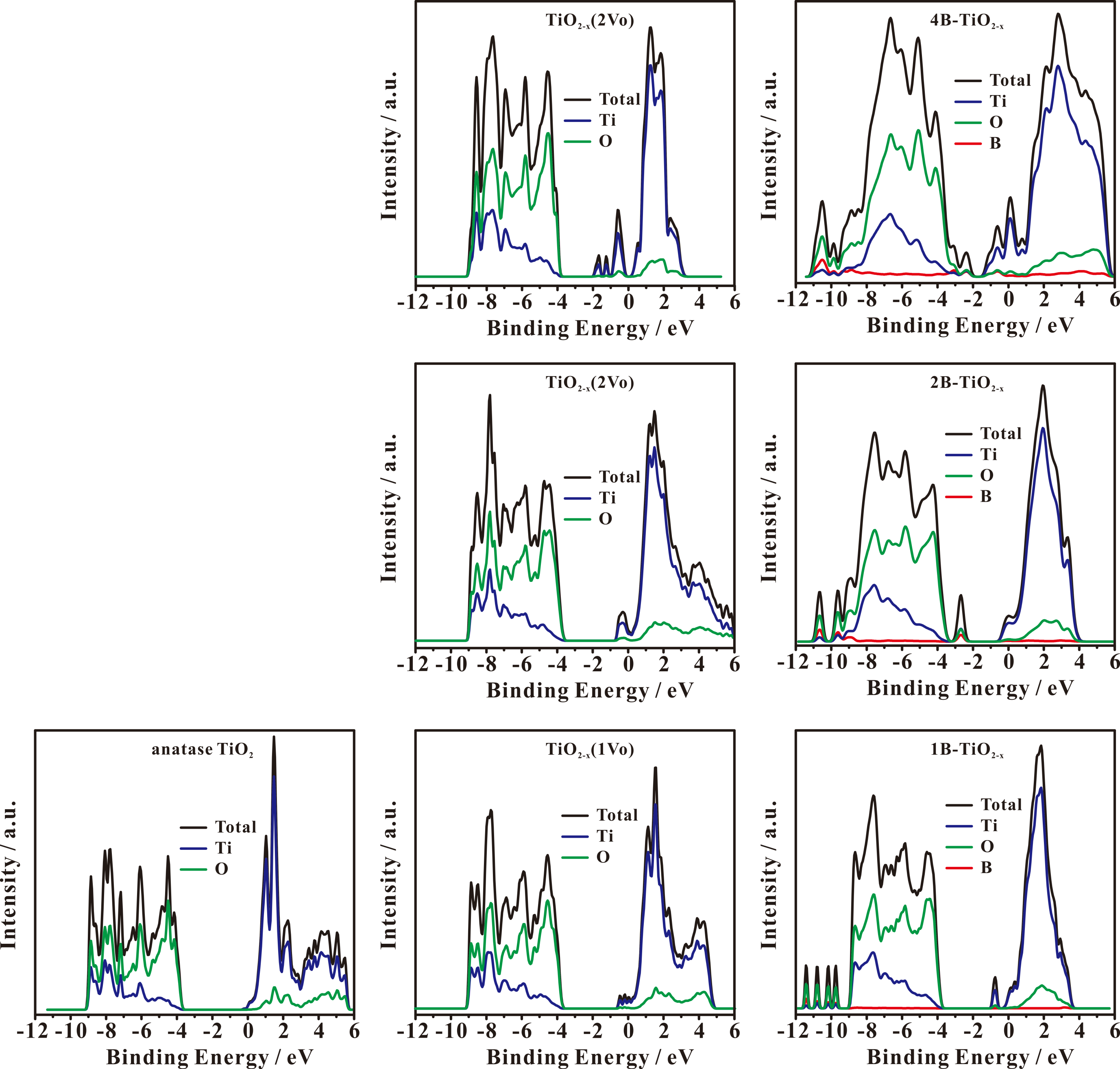


**Figure S6.** Calculated total and project DOS of anatase TiO_2_, various TiO_2-x_ with variable oxygen vacancy (Vo) and various B-TiO_2-x_ with variable B doping.

**References:**

1. Massiot, D. *et al.* Modelling one- and two-dimensional solid-state NMR spectra. *Magn. Reson. Chem.* **40**, 70–76 (2002).

2. Kresse, G. & Furthmuller, J. Efficient iterative schemes for ab initio total-energy calculations using a plane-wave basis set. *Phys. Rev. B* **54**, 11169–11186 (1996).

3. Perdew, J. P., Burke, K. & Ernzerhof, M. Generalized Gradient Approximation Made Simple. *Phys. Rev. Lett.* **77**, 3865–3868 (1996).

4. Kresse, G. & Joubert, D. From ultrasoft pseudopotentials to the projector augmented-wave method. *Phys. Rev. B* **59**, 1758–1775 (1999).

5. Monkhorst, H. J. & Pack, J. D. Special points for Brillouin-zone integrations. *Phys. Rev. B* **13**, 5188–5192 (1976).

6. Burdett, J. K., Hughbanks, T., Miller, G. J., Richardson Jr., J. W. & Smith, J. V. Structural-Electronic Relationships in Inorganic Solids: Powder Neutron Diffraction Studies of the Rutile and Anatase Polymorphs of Titanium Dioxide at 15 and 295 K. *J. Am. Chem. Soc.* **109**, 3639–3664 (1987).

7. Lazzeri, M., Vittadini, A. & Selloni, A. Structure and energetics of stoichiometric TiO2 anatase surfaces. *Phys. Rev. B* **63**, 155409 (2001).

8. Heyd, J., Scuseria, G. E. & Ernzerhof, M. Hybrid functionals based on a screened Coulomb potential. *J. Chem. Phys.* **118**, 8207–8215 (2003).
